# The effect of exercise and physical activity-interventions on step count and intensity level in individuals with multiple sclerosis: a systematic review and meta-analysis of randomized controlled trials

**DOI:** 10.3389/fspor.2023.1162278

**Published:** 2023-07-31

**Authors:** Ellen Christin Arntzen, Razieh Bidhendi-Yarandi, Marianne Sivertsen, Karina Knutsen, Stine Susanne Haakonsen Dahl, Maria Grytvik Hartvedt, Britt Normann, Samira Behboudi-Gandevani

**Affiliations:** ^1^ Faculty of Nursing and Health Sciences, Nord University, Bodø, Norway; ^2^ Department of Biostatistics, University of Social Welfare and Rehabilitation Sciences, Tehran, Iran; ^3^ Physiotherapy Department, Nordland Hospital Trust, Bodø, Norway

**Keywords:** exercise, physiotherapy, multiple sclerosis, physical activity, steps-count

## Abstract

**Background:**

Reduced physical activity is a worldwide challenge in individuals with multiple sclerosis (MS). The aim of this systematic review and meta-analysis was to identify devise-measured effects of physical activity, exercise and physiotherapy-interventions on step count and intensity level of physical activity in individuals with MS.

**Methods:**

A systematic search of the databases of PubMed (including Medline), Scopus, CINHAL and Web of Science was carried out to retrieve studies published in the English language from the inception to the first of May 2023. All trials concerning the effectiveness of different types of exercise on step count and intensity level in people with MS were included. The quality of the included studies and their risk of bias were critically appraised using The modified consolidated standards of reporting trials and the Cochrane Risk of Bias tool, respectively. The pooled standardized mean difference (SMD) and 95% CI of the step-count outcome and moderate to vigorous intensity level before versus after treatment were estimated in both Intervention and Control groups using the random effect model. The Harbord test were used to account for heterogeneity between studies and assess publication bias, respectively. Further sensitivity analysis helped with the verification of the reliability and stability of our review results.

**Results:**

A total of 8 randomized clinical trials (involving 919 individuals with MS) were included. The participants (including 715 (77.8%) female and 204 (22.2%) male) had been randomly assigned to the Intervention (*n *= 493) or Control group (*n *= 426). The pooled mean (95% CI) age and BMI of participants were 49.4 years (95% CI: 47.4, 51.4 years) and 27.7 kg/m^2^ (95% CI: 26.4, 29 kg/m^2^), respectively. In terms of the comparison within the Intervention and the Control groups before and after the intervention, the results of the meta-analysis indicate that the pooled standardized mean difference (SMD) for step-count in the Intervention group was 0.56 (95% CI: -0.42, 1.54), while in the Control group it was 0.12 (95% CI: -0.05, 0.28). Furthermore, there was no significant difference in the pooled SMD of step-count in the physical activity Intervention group compared to the Controls after the intervention (pooled standard mean difference = 0.19, 95% CI: -0.36,0.74). Subgroup analysis on moderate to vigorous intensity level of physical activity revealed no significant effect of the physical activity intervention in the Intervention group compared to the Control group after the intervention, or within groups before and after the intervention. Results of meta regression showed that age, BMI, duration of disease and Expanded Disability Status Scale (EDSS) score were not the potential sources of heterogeneity (all *p* > 0.05). Data on the potential harms of the interventions were limited.

**Conclusion:**

The results of this meta-analysis showed no significant differences in step count and moderate to vigorous physical activity level among individuals with MS, both within and between groups receiving physical activity interventions. More studies that objectively measure physical activity are needed.

**Systematic Review Registration:**

https://www.crd.york.ac.uk/prospero/, identifier: CRD42022343621

## Introduction

Reduced physical activity is a worldwide challenge in individuals with multiple sclerosis (MS), also when disability is minor (1, 2). Typically, this chronic, neurological disease is accompanied by a variety of sensory, motor, visual and cognitive symptoms as well as fatigue (3–5), which all may influence physical activity negatively. MS guidelines advise starting personalized physical activity and exercise when disability is low to prevent the accumulation of impairments and to decrease symptoms (6–10). The negative effects of decreased physical activity are increased risk of comorbidities such as cardiovascular diseases, diabetes, depression, and fatigue, as well as exacerbation of difficulties with mobility and balance in individuals with MS (7, 10).

Physical activity is defined as any bodily movement produced by skeletal muscles that requires energy expenditure (11). The concept refers to all movement, including walking. Extensive evidence exists for the benefits of physical activity, exercises, and physiotherapy, such as reduction of fatigue (12, 13), improvement of balance and walking (14), better health related quality of life (HRQoL) (14), and improved neuromuscular and physical functioning in individuals with MS (7). Recent studies indicate that physical activity may potentially modify the disease (9, 15). Even so, a report demonstrate that only approximately 20% of individuals with MS meet the general and MS specific physical activity recommendations (16) of a minimum of 150 min of exercise of lifestyle physical activity per week (30 min per week-day) (10). This is supported by a meta-analysis documenting a daily mean step count of 5,840 (SD 3,096) and mean minutes of moderate to vigorous physical activity per day of 18.4 (SD: 21.1), which suggest that people with MS are less active than a general population (1). A recent multi-national study report that the problem increased during the Covid-19 pandemic (17), where a high percentage of respondents who were active before the pandemic but not during it, surprisingly, had little intention of changing this negative trend (18). This calls for action, and a need to explore physical activity interventions particularly in terms of step count and intensity levels.

It is challenging to increase physical activity, regardless of whether interventions focus on exercise, behavioral change or a mix between the two (19). A recent systematic review addressing physical activity behavior-interventions demonstrated no change in objective measures or long-term physical activity (19), while subjective measures did improve (19). Another systematic review and meta-analysis found that behavioral change interventions alone demonstrated a moderate effect [standardized mean difference (SMD) = 0.71] with a medium level of heterogeneity (*I*^2^ = 54%) for changing physical activity levels in individuals with MS (20). Interventions mixing behavioral change techniques and exercises showed an SMD of 0.38 and interventions containing only exercises demonstrated 0.53 with medium levels of heterogeneity (*I*^2 ^= 63%) (20). The most commonly used tools to examine physical activity objectively are Uni-axial accelerometers, pedometers and multi-sensor systems (21). The outputs of these monitors are activity counts and steps per day, as well as minutes of light, moderate and vigorous physical activity per day (21). Studies using such monitors for measuring physical activity can provide objective knowledge of the potential effectiveness of various exercise, physiotherapy and physical activity interventions.

Besides, A meta-analysis serves as a valuable tool in evidence synthesis, enabling a comprehensive evaluation of research findings by systematically integrating data from multiple independent studies. This approach offers several key benefits. It could provide a more precise estimate of the treatment effect, enhances statistical power, and offers a comprehensive overview of the research landscape. Additionally, it enhances generalizability and external validity by examining results across diverse populations, and settings, thereby supporting informed clinical practice and policy decisions. However, pooling data from multiple studies in a meta-analysis increases statistical power, allowing for the detection of smaller treatment effects that may not be apparent in individual studies alone. Overall, a meta-analysis offers a robust approach to synthesizing evidence and can significantly contribute to advancing knowledge in the field. Therefore, the aim of this systematic review and meta-analysis was to identify the effects of physical activity, exercise and physiotherapy-interventions on step count and intensity of physical activity in individuals with MS.

## Methods

This systematic review and meta-analysis was conducted based on the recommendations from Cochrane and is reported according to the Preferred Reporting Items for Systematic Reviews and Meta-Analyses (PRISMA) statement (22, 23). The study protocol holds a detailed description of the study and has been registered in the PROSPERO database (PROSPERO CRD42022343621). The review question was framed using the PICO (Population, Intervention, Control, and Outcomes) statement as follows: P: male and female individuals clinically diagnosed with MS; I: Different types of physical activity; C: participants undergoing usual practice or no exercise; O: step-count which measured by the number of steps per day and intensity of physical activity.

### Eligibility criteria and outcome of study

Criteria for the inclusion/exclusion of studies were established prior to the literature search. Studies had to fulfill the following criteria for eligibility: (i) English-language peer-reviewed publication; (ii) individuals with a confirmed diagnosis of MS based on validated criteria (24), or physician's confirmation (iii) randomized trial with allocation to either a physical activity, exercise or physiotherapy intervention at any frequency, duration, and intensity or a control condition undergoing usual practice or active treatment; (iv) reporting the mean (SD) of step-counts per day and also physical activity levels. Likewise, studies without accurate or clear data, incomplete results or data not provided in the required format for the pooled analysis of research variables, gray literature and non-original studies including reviews, commentaries, editorials, letters, meeting abstracts, case reports, conference proceedings, dissertations, theses, unpublished data and presentations were excluded. Moreover, we excluded studies that included participants with significant co-morbidities for which exercise would be contra-indicated such as unstable cardiovascular conditions (e.g., recent heart attack, severe heart failure). Additionally, multiple reports from the same trial were combined into a single unit of analysis.

The primary outcome of interest was step-count, which was defined as the number of steps taken per day. The secondary outcome of the study was intensity of physical activity, which is often measured as minutes of inactivity as well as minutes of low, moderate and high intensity. Common descriptions of the various intensities are: inactive; lying or sitting still, low; for example standing, cooking, playing the piano or exercises as light walking and stretching, moderate intensity; for example stair climbing, lifting children or washing the car or exercises such as tennis and water aerobics, vigorous; such as heavy gardening or exercise such as jogging, fast dancing or soccer. When measuring intensity with an accelerometer the cut-off points for defining each level may vary and some studies report each level separately while others collapse the moderate and vigorous levels. We examined the outcomes of interest at the post-intervention follow-up. Some studies had additional long term follow-ups after the intervention, however, as there were not enough data to include in the meta-analysis, these were excluded.

### Search strategy

A comprehensive literature search was conducted on the databases of PubMed (including Medline), Scopus, CINHAL and Web of Science to retrieve studies published in the English language from the inception to first of May 2023. A search strategy with various synonyms was entered as free-text terms in those databases in an attempt to maximize the sensitivity of the search strategy. Further, a manual hand search in the references list of selected studies and other relevant reviews was carried out to maximize the identification of eligible studies. The keywords, alone or in combination, were used during the search process are presented in Supplementary Table S1.

### Study selection and data extraction

The titles, abstracts, and full texts of selected studies were screened independently by four reviewers (KK, SSHD, MS, BN and MGH). Any disagreement was resolved by discussion with senior authors (ECA and SB-G). The following data was extracted from eligible studies: first author's name; publication year; country; study design; sample size; population demographic details including age, body mass index (BMI) and gender; disease characteristics including Expanded Disability Status Scale (EDSS) and years since diagnosis; intervention details; follow-up length and outcome measures. The data extraction process was double-checked to ensure the accuracy of data collection before the meta-analysis and prevent bias in data extraction and data entry. Moreover, in case of missing data or ambiguities in study design or trial conduction, the study authors were contacted by e-mail to request additional information, however we did not get response from the authors.

### Quality appraisal

The quality of the included studies was critically appraised in terms of the methodological structure and presentation of findings. The modified consolidated standards of reporting trials (CONSORT) as a validated quality assessment. Checklist for RCTs was used for the appraisal. A study can be awarded >75% of the highest score of the CONSORT checklist and judged as high quality, >50%–75% as moderate quality, >25%–50% as low quality, and 25% or less as very low quality (25). The risk of bias in these studies was assessed using the Cochrane Collaboration tool for assessing the risk of bias for RCTs (26). Accordingly, the risk of bias was categorized as ‘low risk’, ‘high risk’, and ‘unclear risk’.

### Statistical analysis

All statistical analysis was performed using STATA software (version 14; STATA, INC., College Station, TX, USA). Heterogeneity between the studies was assessed using the I^2^ index and chi-squared test, *I*^2^ > 50% and *P* < 0.05 were interpreted as heterogeneity. Heterogeneous and non-heterogeneous results were analyzed using the random and fixed effects models for calculating the pooled effect, respectively. Publication bias was assessed using a funnel plot. Pooled SMD and 95% CI of the step-count outcome before versus after treatment were estimated in both Intervention and Control groups using the random effect model. Pooled SMD and 95% CI of the step-count in treated groups versus controls were also estimated by the random effect model. A meta-regression analysis was run to assess the age, BMI, duration of disease and the EDSS score as the potential sources of heterogeneity. Sensitivity analysis (leave-one out) was run to investigate the influence of a single individual study on the pooled results. Significant level was considered *p*-value < 0.05.

## Results

**Figure 1:**
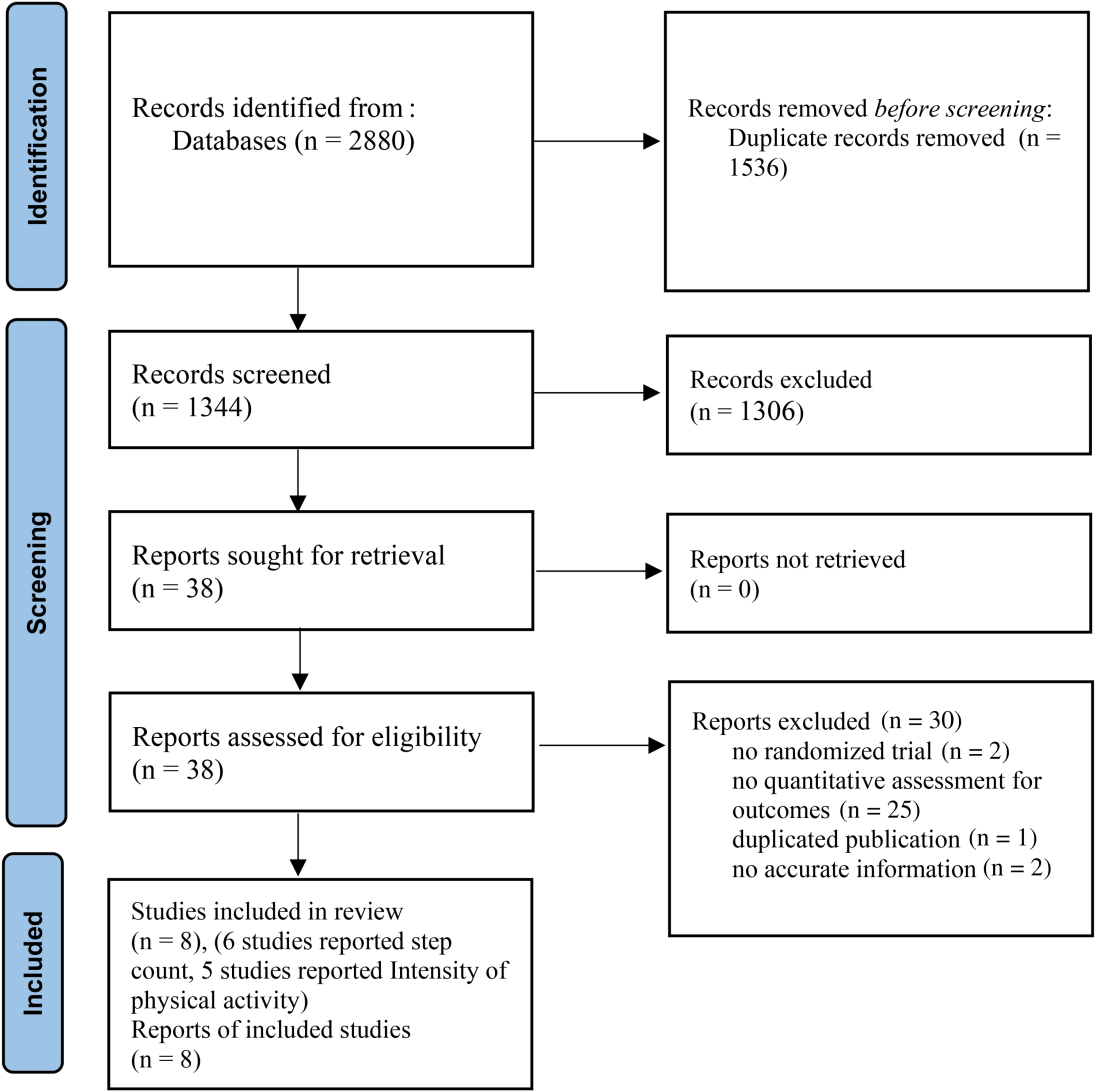
PRISMA flow diagram of the selection process.

The initial literature search yielded 2,880 studies, 38 of which were further evaluated by retrieving their full text and 30 of these were excluded. Eventually, 8 eligible studies were included in the systematic review and offered extractable data for the meta-analysis (2, 27–33). A flow diagram of this process is presented in Figure 1.

The main characteristics of the studies included are summarized in Table 1. All studies had the randomized design, and in all RCTs physical activity was devise measured. Intervention types were as follows: Six interventions focused on information and behavioral change strategies delivered in various ways. Plow and colleges (2019) examined an intervention that consisted of six group telephone calls and four individually tailored calls through 12 weeks addressing physical activity compared to a similar intervention also including fatigue management (30). Nasseri and colleges (2020) explored an intervention that lasted for 12 weeks where the participants had access to an evidence-based patient information app (EBPI), compared to a control group which received general information on the positive health benefits of exercise (29). Pilutti and colleges (2014) examined an internet delivered intervention containing 15 web-based video coaching sessions led by a behavioral coach with the goal to increase lifestyle physical activity, primarily walking, compared with a control group (33). Motl et al. (2017) conducted a six month long intervention addressing behavioral change through a web site and individual video-chats with a behavioral coach compared to wait-list controls (27). Motl et al. (2023) additionally compared a six month long web-based intervention using e-learning approaches based on social cognitive theory with a control group receiving attention/social contact (31). Learmonth and colleges (2016) examined a four month home-based exercise training program based on physical activity guidelines and supplemented by behavioral change strategies compared to wait-list controls (31) Paul and colleges (2019) examined an intervention with 48 sessions throughout 24 weeks of home-based physiotherapy delivered through a web site and compared to individuals receiving a sheet of exercises (28). Only one intervention provided a face-to-face setting for conducting exercises together with a physiotherapist (2). This intervention examined by Arntzen and colleges (2020) comprised of 18 sessions throughout six weeks, was performed in groups of three led by a physiotherapist and compared to usual care (2).

**Table 1:** Characteristics of the randomized controlled trials included in the final meta-analyses.

Study Characteristics			Population Characteristics		Intervention Characteristics					Outcome of study			
					Case				Control	Intervention Group		Control Group	
Author, year	Country	Study design	Intervention Group Characteristics	Control Group Characteristics	Training Regimen	Duration (wk)	Frequency (x/wk)	Time min		Step count, per day	Intensity	Step count, per day	Intensity
Arntzen et al. (2020)	Norway	Assessor-blinded prospective RCT	SS: 39 Age: 52.2 (12.9) BMI: NM % Women: 69.2% EDSS: 2.45 (1.65) Disease Duration: 10.0 (7.8)	SS: 40 Age: 48 (8.75) BMI: NM % Women: 72.5% EDSS: 2.28 (1.28) Disease Duration: 10.6 (7.2)	Group-based, individualized, comprehensive core stability and balance intervention (GroupCoreDIST)	6	3	60 min	Standard care	Baseline: 6,454.33 (3,856.16) After intervention: 6,562.26 (3,458.89)	Baseline: 31.14 (30.37) After intervention: 23.51 (21.54)	Baseline: 6,467.50 (3,366.67) After intervention: 6,598.23 (3,797.50)	Baseline: 26.21 (26.59) After intervention: 20.24 (19.39)
Nasseri et al. (2020)	Germany	Rater-blinded RCT	SS: 18 Age: 49.6 (8.5) BMI: NM % Women: 50% EDSS: 3.5 (2.5–6.0)* Disease Duration: 13.1 (5.6)	SS: 20 Age: 52.5 (7.3) BMI: NM % Women: 55% EDSS: 3.5 (3.0–6.0)* Disease Duration: 20.1 (13.0)	Access to multimedia evidence-based patient information (EBPI) app including activity feedback, texts, figures and videos	12	NM	NM	Access to very simple two page leaflet with general information about the health effects of exercising without any EBPI content	Baseline: 7.3 (2.8)^€^ After intervention: 6.7 (2.7)^€^	–	Baseline: 5.8 (2.3)^€^ After intervention: 7.0 (2.4)^€^	–
Plow et al. (2019)	USA	Single-blinded RCT	*Group 1:* SS: 69 Age: 51.2 (9.2) BMI: NM % Women: 79.7% EDSS: NM Disease Duration: NM *Group 2:* SS: 70 Age: 53.2 (6.5) BMI: NM % Women: 90% EDSS: NM Disease Duration: NM	SS: 69 Age: 51.8 (9.3) BMI: NM % Women: 84.1% EDSS: Disease Duration: NM	*Group 1:* Physical activity only intervention *Group 2:* Physical activity plus fatigue self-management intervention	12	NM	NM	Contact-control intervention	*Group 1:* Baseline: 4,032.69 (2,229.75) After intervention: 4,635.67 (2,722.15) *Group 2:* Baseline: 3,992.70 (2,166.04) After intervention: 4,124.80 (2,118.92)	*Group 1:* Baseline: 15.71 (15.91) After intervention: 24.39 (27.12) *Group 2:* Baseline: 18.30 (41.56) After intervention: 16.07 (12.38)	Baseline: 3,599.26 (2,255.66) After intervention: 3,537.64 (2,050.88)	Baseline: 10.75 (12.98)After intervention: 10.40 (10.81)
Motl et al. (2017)	USA	RCT	SS: 23 Age: 52.3 (10.3) BMI: 29.1 (7.5) % Women: 91.3% EDSS: 3.5 (1.5) Disease Duration: 14.4 (10.4)	SS: 24 Age: 51.4 (7.4) BMI: 29.0 (7.4) % Women: 79.1% EDSS: 3.5 (2.0) Disease Duration: 12.1 (8.7)	Behavioral intervention consisted of two primary components, namely a dedicated Internet website and one-on-one video chats with a behavioral coach	24	NM	NM	Usual care	Baseline: 3,365 (2,125) After intervention: 6,095 (4,125)	Baseline: 13.9 (9.2) After intervention: 28.8 (5.0)	Baseline: - After intervention: –	Baseline: 17.5 (18.3) After intervention: 20.2 (5.2)
Paul et al. (2019)	UK	Multi-centre, RCT	SS: 45 Age: 55.6 (10.2) BMI: 25.8 (5.1) % Women: 71.1% EDSS: 6.0 (6–6)* Disease Duration: 10 (6–18)*	SS: 45 Age: 56.5 (9.1) BMI: 26.4 (5.6) % Women: 82.2% EDSS: 6.0 (6–6)* Disease Duration: 15 (10–23)*	Home exercise program delivered via web-based physiotherapy	24	2	NM	Sheet of exercises	Baseline: 4,451 (2,511) After intervention: 4,017 (2,493)	–	Baseline: 4,584 (2,788) After intervention: 4,271 (2,272)	–
Motl et al. (2023)	USA	parallel group RCT	SS: 159 Age: 48.8 (9.4) BMI: – % Women: 90.4% EDSS: – Disease Duration: 13.9 (8.7)	SS: 159 Age: 48.8 (9.5) BMI: – % Women: 86.5% EDSS: – Disease Duration: 11.4 (8.8)	Behavioral intervention based on social cognitive theory and delivered through internet using e-learning approaches	24			“Wellness for MS” (attention/social contact)	Baseline: 4,035 (207) After intervention: 4,683 (212)	Baseline: 15.8 (1.5) After intervention: 21.7 (1.5)	Baseline: 3,719 (208) After intervention: 3,874 (210)	Baseline: 13.3 (1.5) After intervention: 14.5 (1.5)
Learmonth et al. (2017)	Australia	RCT	SS: 29 Age: 48.7 (10.4) BMI: – % Women: 96.5% EDSS: 1.25 (0–6, 2.5)* Disease Duration: 14.8 (8.5)	SS: 28 Age: 48.2 (9.1) BMI: – % Women: 96.4% EDSS: 2 (0–5.5, 3)* Disease Duration: 13.0 (7.7)	Home-based exercise program based on recent PA guidelines for MS, supplemented by behavioral change strategies: aerobic training × 2 per week, 10−30 min Strength training × 2 per week	16	4		Wait list	–	Baseline: 25.54 (18.05) After intervention: 26.64 (15.49)	–	Baseline: 21.05 (18.78) After intervention: 22.55 (22.46)
Pilutti et al. (2014)	USA	RCT	SS: 41 Age: 48.4 (9.1) BMI: – % Women: 71.1% EDSS: – Disease Duration: 10.6 (7.1)	SS: 41 Age: 49.5 (9.2) BMI: – % Women: 78% EDSS: – Disease Duration: 13.0 (9.1)	Web-based behavioral intervention to increase lifestyle physical activity, primarily walking. 15 web-based video coaching sessions	24				–	Baseline: 17.0 (22.4) After intervention: 19.5 (2.3)	–	Baseline: 16.2 (17.7) After intervention: 13.8 (2.2)

EDSS, expanded disability status scale; RCT, randomized controlled trial; NM, not mentioned; SS, sample size; BMI, body mass index.

* Median (IQR).

^€^ Per minute.

The length of the follow-ups in the studies ranged from 6 to 24 weeks. The studies were conducted in USA (27, 30, 31, 33), Norway (2), UK (28), Australia (32) and Germany (29) and were published in recent 10 years between 2014–2023.

Six articles reported the step count (2, 27–31) and 5 articles (2, 27, 31–33) presented the intensity of physical activity at moderate to vigorous level.

There was a total of 919 participants with MS [715 (77.8%) female and 204 (22.2%) male] randomly assigned to Intervention (*n *= 493) or Controls (*n *= 426). The pooled mean (95% CI) age and BMI of participants were 49.4 years (95% CI: 47.4, 51.4 years) and 27.7 kg/m^2^ (95% CI: 26.4, 29 kg/m^2^), respectively. In addition, the pooled mean (95% CI) of disease duration and EDSS score in participants were 12.3 years (95% CI: 11.2, 13.4 years) and 2.9 (95% CI: 2.6, 3.2), respectively.

**Figure 2:**
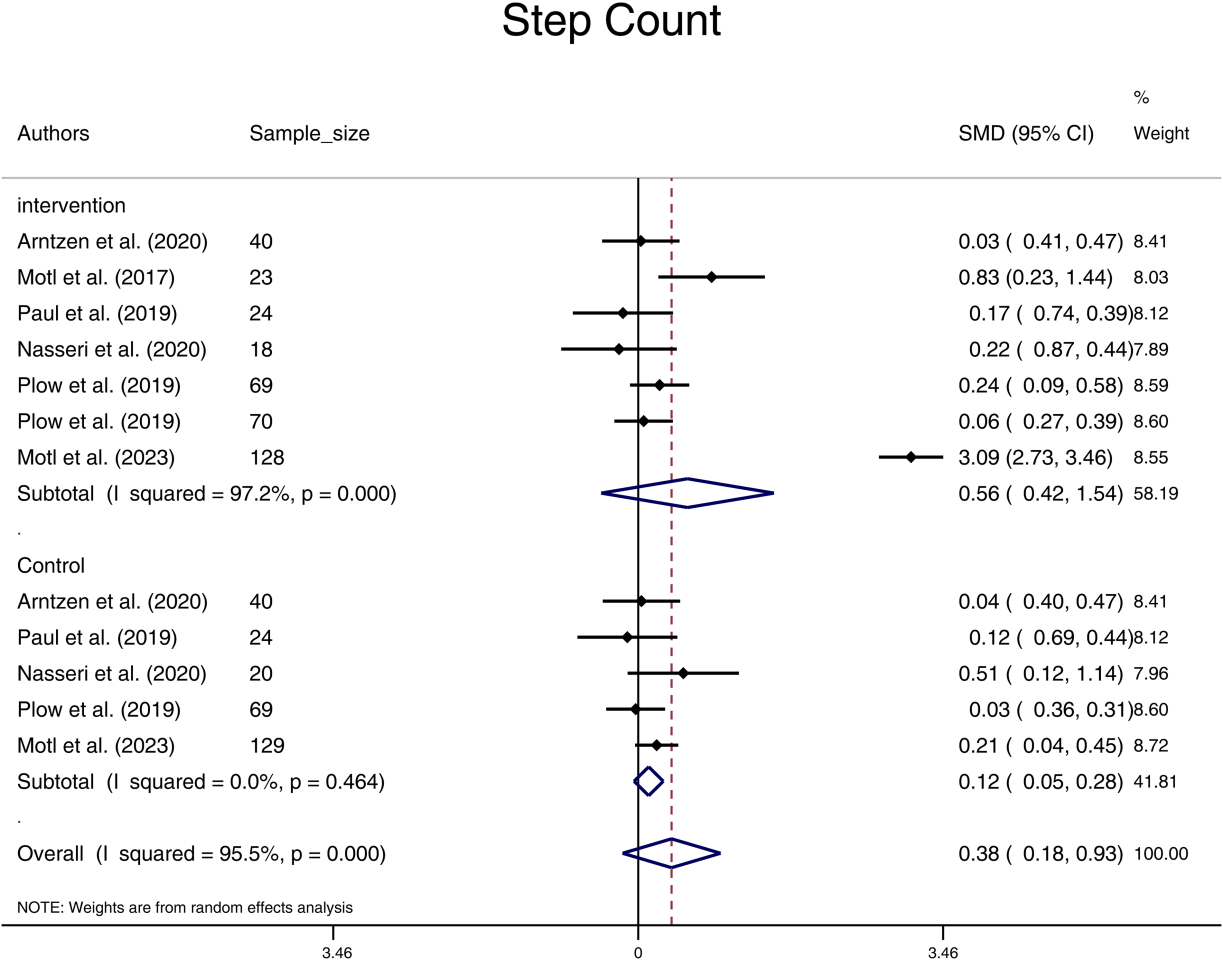
Forest plot of step count pooled standard mean difference before and after physical activity intervention in intervention and control groups. (SMD, standardized mean difference; CI, confidence interval).

In terms of the comparison within the Intervention and the Control groups before and after the intervention, the results of the meta-analysis indicate that the pooled standardized mean difference (SMD) for step-count in the Intervention group was 0.56 (95% CI: -0.42, 1.54), while in the Control group it was 0.12 (95% CI: -0.05, 0.28). These findings suggest that there were no statistically significant differences in the SMD of step-count before and after the physical activity/exercise/physiotherapy intervention in both groups (Figure 2). There was no significant heterogeneity among the studies in the control group (*I*^2^ value of 0%, *p* = 0.464). However, significant heterogeneity was observed among the studies in the Intervention groups (*I*^2^ value of 97.2%, (*p* < 0.001).

**Figure 3:**
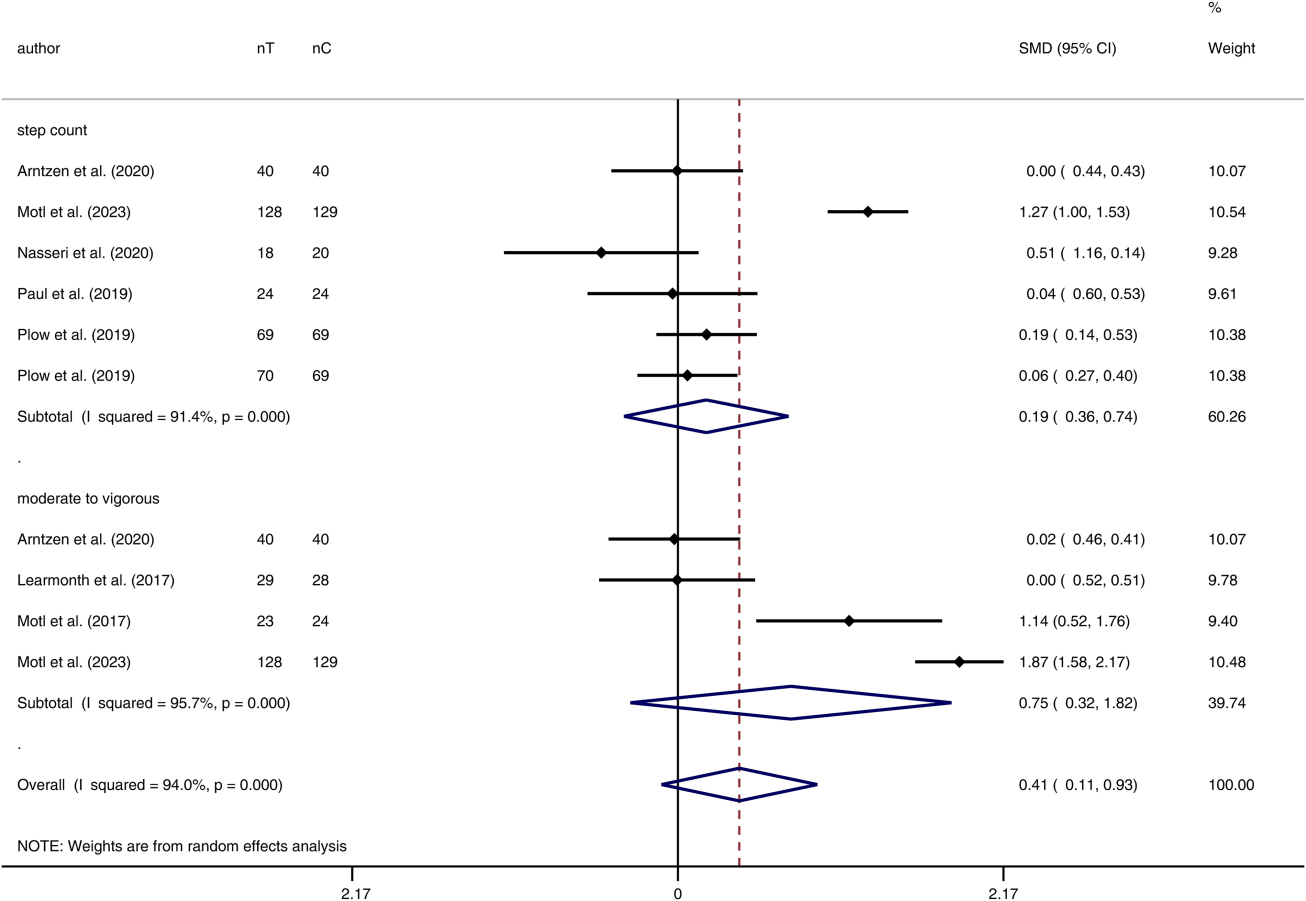
Forest plot of step count and intensity level of physical activity pooled standard mean difference in intervention group versus control group after the intervention. (nT, number of samples in treatment group; nC, number of samples in control group; SMD, standardized mean difference; CI, confidence interval).

In terms of comparison between groups after the physical activity intervention, the results showed that there was no significant difference in the pooled standard mean difference of step-count in the Intervention group compared to the Controls (pooled standard mean difference = 0.19, 95% CI: -0.36,0.74), (Figure 3). Substantial heterogeneity was found between studies (*I*^2 ^= 91.4%; *p *< 0.001).

**Figure 4:**
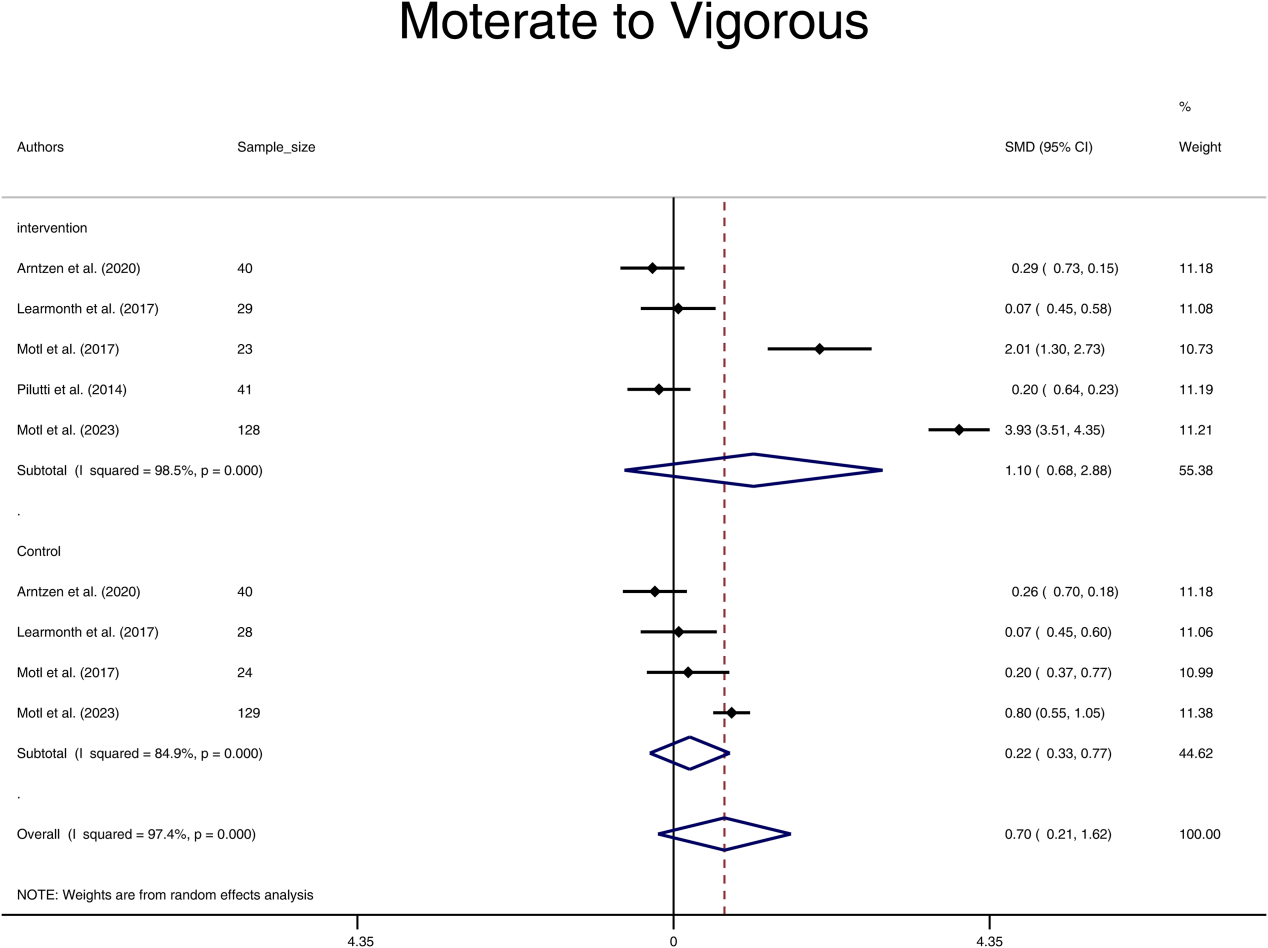
Forest plot of moderate to vigorous pooled standard mean difference before and after physical activity intervention in intervention and control groups. (SMD, standardized mean difference; CI, confidence interval).

With limited studies reporting the intensity of physical activity at some levels, we were underpowered in the evaluation of subgroups of light, moderate, and vigorous physical activity. Subgroup analysis on moderate to vigorous physical activity intensity revealed no significant effect of the physical activity intervention in the Intervention group compared to the Control group after the intervention (SMD = 0.75, 95% CI: -0.32, 1.82) (Figure 3). Additionally, there were no significant differences in moderate to vigorous physical activity levels before and after the intervention in both groups, with SMD = 1.10 (95% CI: -0.68, 2.88) for the Intervention group and SMD = 0.22 (95% CI: -0.33, 0.77) for the Control group (Figure 4). On evaluation, significant heterogeneity was observed among the studies included in these analyses (all *p *< 0.001).

Results of meta regression showed that age, BMI, duration of disease and EDSS score were not the potential sources of heterogeneity in both outcomes of step count and moderate to vigorous physical activity (all *p *> 0.05) (Supplementary Figure S1).

Data on potential harms of the intervention were limited.

### Quality assessment, risk of bias, publication bias and sensitivity analyses

**Figure 5:**
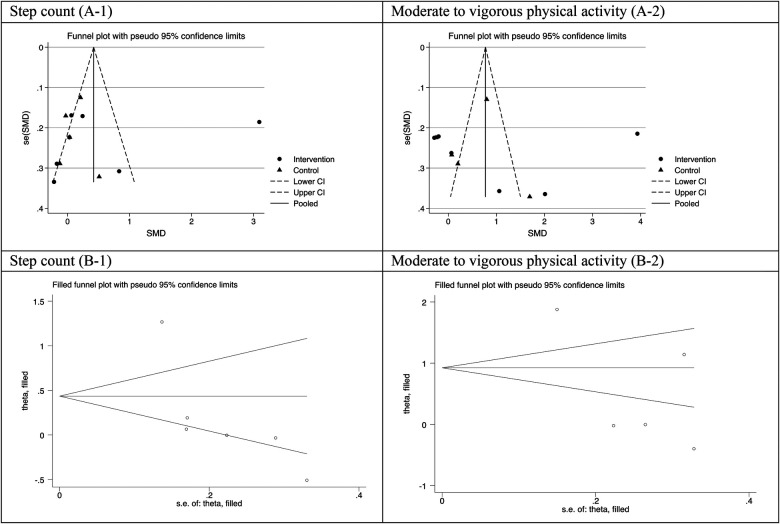
Funnel plot (**A-1,A-2**) and filled funnel plot (**B-1,B-2**) for assessing publication bias step count and moderate to vigorous intensity of physical activity.

**Figure 6:**
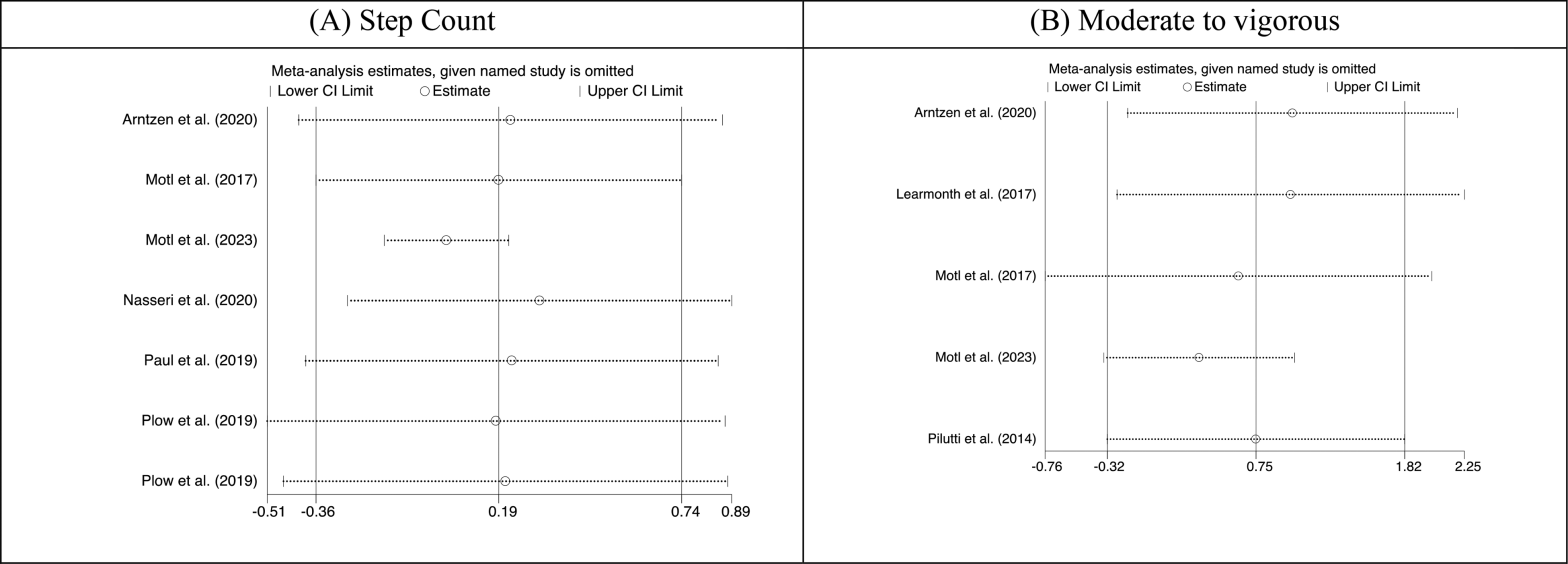
Plot of sensitivity analysis result for (**A**) step count and (**B**) moderate to vigorous physical activity.

Visual inspection of the funnel plot for step-count and moderate to vigorous intensity of physical activity outcomes were largely symmetrical, suggesting a low risk of publication bias, which was supported by Egger test (all *P* value > 0.05) (Figure 5). The results were highly consistent with the main results of data analysis, and no substantial modification of the estimates change was reported after the exclusion of individual studies (Figure 6).

The quality assessment of the included studies is presented in Supplementary Table S2. A total of 38% studies were classified as high quality (2, 28, 31), 62% as moderate quality (27, 29, 30, 32, 33), but no study had low or very low quality. Assessment of risk of bias levels of key RCT components including bias in random sequence generation, allocation concealment, blinding of participants, personnel, and outcome assessment, incomplete outcome data and selective outcome reporting showed that generally all studies had low risk of bias in all items, only 12,5% had unclear risk of bias in two items of random sequence generation, allocation concealment (Supplementary Figures S2A, B).

## Discussion

The aim of this systematic review and meta-analysis was to identify the effects of physical activity, exercise and physiotherapy-interventions on step count and intensity levels of physical activity in individuals with MS. The results demonstrated no significant difference between or within Interventions and Control groups, neither in terms of number of steps nor minutes per day of moderate to vigorous physical activity. Six of the included studies measured number of steps, and five studies reported on moderate to vigorous physical activity levels. Only three of the papers used number of steps or intensity level as a primary outcome (27, 31, 33). The other studies used devised physical activity as secondary outcomes (2, 28–30, 32) which indicate that these elements were not primarily aimed to improve through the intervention. This may be one explanation for the lack of significant change in physical activity demonstrated in the current study.

Looking at the studies separately, the studies by Motl (27, 31), where the participants were followed for 6 months, demonstrated significant changes. This may indicate that a long term follow-up emphasizing behavioral change over time is needed to improve physical activity. Pillutti (33) also had a six month follow-up and demonstrated tendencies for increased moderate to vigorous physical activity, although not reaching significance. Plow (30) did, however, also demonstrate significant change in the physical activity group compared to a control group after a 12 week long intervention. The optimal length for physical activity interventions seems unclear and should be explored further.

Regarding the content of the interventions, seven studies emphasized internet delivered interventions, using information and behavioral change strategies through telephone calls (30), an information app (29), websites (27, 31, 33) and two studies added web-based home exercises (28, 32). Only one intervention provided a face-to-face approach, conducting group-based exercises and encouraging for home exercises (2). The various web-based solutions addressed in the included papers can be a good opportunity to enhance a sustainable long-term follow-up of persons with MS. This is important as only half of those with mild disability, and the majority of those with moderate and severe disability report not meeting the guidelines for physical activity (16, 18). To enhance sustained adequate levels of physical activity, a blended physical and digitally supported follow-up is suggested (34). This is in line with individuals with MS′ wishes, as many report to prefer a face-to-face setting and few report to prefer only digital follow-up (18). Facilitators and barriers towards being adequately physically active need further attention since MS is a complex disease and both symptoms and disease progression are highly individual (20). Studies with high user involvement addressing what engages individuals with MS to become and stay physically active are warranted.

Walking is identified as the most commonly used activity in individuals with MS (18). This is an available activity for many individuals with MS, since a high proportion of the MS population have mild to moderate disability (EDSS 0–4) (35). The existing walking-interventions often focus on reducing fatigue (36, 37), cardiovascular parameters (36) and quality of life (38). There is a need for creating new interventions that include walking, also at fast pace or even running, as this may increase both number of steps and the intensity level of physical activity. We should, however, be mindful that impairments such as somatosensory problems, paresis, visual problems, postural control and balance problems appear even when disability is low (39, 40). Interventions that integrate a personalized focus on the prerequisites for walking as well as the activity itself may be of interest. In this regard, one of the studies in our current meta-analysis demonstrated long-term significant between-group changes in both balance and walking, after only six weeks of group-based exercises focusing on prerequisites for balance (2). Such positive changes is often followed by users experiencing new affordances for daily activities from change in sensorimotor function (41), which may be a good basis for motivation to behavioral change in physical activity in a population with chronic and progressing symptoms.

There is extensive evidence for the benefits of physical activity both to reduce fatigue (12, 13) the risk of falling (3, 4, 42, 43), and improve balance and walking (14), neuromuscular and physical functioning (7), as well as HRQoL (14). In this respect, it is a paradox that physical activity is not systematically addressed or measured in the follow-up of individuals with MS throughout the disease course. When exploring physical activity, one should keep in mind that activities such as biking, rowing, Pilates, yoga and strength training may not increase acceleration or number of steps. Future studies may additionally report pulse, since such recordings may inform us regarding intensity of physical activity during activities without acceleration. Quantification of the subjective experience of physical activity is a good supplement, as there are many studies demonstrating that individuals with MS experience possibilities for increased physical activity from several interventions (27, 32, 33).

Our study was limited by a number of factors that should be considered in the interpretation of data. Reporting physical activity interventions has inconsistencies and inadequacies, affecting evidence synthesis and strengthening the need for standardization. Although a standardized definition was applied for step-count, the definitions and criteria for measuring intensity of physical activity varied among studies. The included studies had appropriate sample sizes in case and control groups and mainly presented a low risk of bias RCTs, but the number of studies assessing the outcomes of interest was limited. It also hindered us to run the subgroup based on a different form of physical activity intervention. The bulk of participants in published clinical trials was patients with mild to moderate disability due to MS. Future studies evaluating persons with more severe disability are needed. Furthermore, all included studies were conducted in high income countries, consequently, the external validity of our results is limited to high income countries.

It should be noted that funnel plot of this meta-analysis seems to be asymmetric. A funnel plot is a graphical representation of the relationship between the effect size estimates from individual studies and their corresponding standard errors. While an asymmetrical plot in a meta-analysis funnel plot may raise concerns about publication bias, it is not definitive evidence, particularly in meta-analysis of RCTs. Various factors, including heterogeneity, small study effects, chance, small number of studies included can contribute to asymmetry (44). In the current study, we applied other sources to check the risk of publication bias as well. The results of Egger test showed that there is not significant publication bias.

There were significant heterogeneity among studies included in most of analysis. However, meta-regression analysis showed that characteristics of the study population age, BMI, disease duration and EDSS score were not the source of heterogenicities. We hypothesized that variation in intervention characteristics in terms of type, intensity, duration, and frequency of interventions across studies, differences in the settings or contexts in which the studies were conducted, time-related factors since studies conducted over different time periods, unmeasured confounding factors such as lifestyle factors may lead to heterogeneity.

## Conclusion

The results of the meta-analysis showed no significant differences in step count and moderate to vigorous physical activity among individuals with MS, both within and between groups receiving physical activity interventions. Even if some of the included studies separately demonstrated significant change in physical activity, the current meta-analysis did not show evidence to support the effectiveness of physical activity, physiotherapy or exercise interventions in both outcomes. This may be due to the lack of studies assessing both step count and intensity of physical activity, therefore this assumption needs to be explored in further studies. There is a need for designing interventions that address and measure number of steps and intensity levels in physical activity systematically in the MS population.

## Data Availability

The original contributions presented in the study are included in the article/**Supplementary Materials**, further inquiries can be directed to the corresponding author.
