# Supplementary material for: The effect of exercise and physical activity-interventions on step count and intensity level in individuals with multiple sclerosis: a systematic review and meta-analysis of randomized controlled trials
Source: Front Sports Act Living. 2023 Jul 31;5:1162278. doi: 10.3389/fspor.2023.1162278 (PMC10425270; doi:10.3389/fspor.2023.1162278)
Supplement: Supplementary file 1 [file Datasheet1.docx]

Supplementary table 1. The specific search strategy will be (taking PubMed as an example):

| Search query | Search keywords (titles, abstracts, general keywords, and subject headings) |
| --- | --- |
| 1 | (“Physical activity”[mh] OR “Physical Therapy”[mh] OR Physical Therapy Modalities[mh] OR Aerobic [tw]OR “Aerobic activity” [mh] OR “Resistance activities” OR Exercise[mh] OR “Exercise therapy”[tw] OR “Therapeutic exercises” [mh] OR Exercise Program[mh] OR “Acute Exercise”[mh] OR “Aerobic Exercise”[mh] OR “Isometric Exercise”[mh] OR “Physical Exercise”[mh] OR “exercise tolerance” [tw] OR Training[mh] OR “resistance training” [mh] OR “Resistance exercises”[tw] OR “Exercise Training”[mh] OR “Balance Training”[tw] OR “Balance exercises”[tw] OR “Endurance Training” [tw] OR “Strength training”[mh] OR “Aquatic exercises”[tw] OR treadmill[tw] OR “treadmill walking” [tw] OR “treadmill training”[tw] OR “treadmill Exercise”[tw] OR physiotherapy[mh] OR “functional therapy” [tw]  OR “Occupational Therapy”[mh] OR Aerobic[mh] OR aquatics[tw] OR Rehabilitation[mh] OR Pilates[mh] OR Stretching[tw] OR “Muscle Stretching Exercises”[mh] OR “Stretching exercises” [mh] OR Yoga[mh] OR “Tai Ji”[mh] OR endurance[tw] OR “Endurance exercises”[tw]) |
| 2 | (“Multiple sclerosis”[mh] OR “Disseminated Sclerosis”[mh] OR MS [mh]) |
| Search query | (1) AND (2) |
| Limitation | English Language, Human studies |

Supplementary Table2. Quality assessment of included studies using the Consort Assessment Scale for interventional studies

| Author | Methods | | | | | | | | | | | | | | | | |
| --- | --- | --- | --- | --- | --- | --- | --- | --- | --- | --- | --- | --- | --- | --- | --- | --- | --- |
|  | Trial design | | Participants | | Interventions | Outcomes | | Sample size | | Randomization | | Allocation concealment mechanism | Implementation | Blinding | | Statistical methods | |
|  | a | b | a | b |  | a | b | a | b | a | b |  |  | a | b | a | b |
| Arntzen et al. (2020) | * | - | * | * | * | * | - | * | * | * | * | * | * | * | * | * | * |
| Nasseri et al. (2020) | * | - | * | * | * | * | - | * | - | * | - | * | - | * | * | * | * |
| Plow et al. (2019) | * | - | * | * | * | * | - | * | - | * | * | * | * | * | - | * | * |
| Motl et al. (2017) | - | - | * | * | * | * | * | - | - | * | - | * | * | - | - | * | * |
| Paul et al. (2019) | - | - | * | * | * | * | - | - | - | * | * | - | * | * | * | * | * |
| Motl et al. (2023) | * | * | * | * | * | * | - | - | - | * | * | * | * | - | - | * | * |
| Learmonth et al. (2017) | * | - | * | * | * | * | - | - | - | * | * | * | * | - | - | * | * |
| Pilutti et al. (2014) | * | - | * | * | * | * | - | - | - | * | * | * | * | - | - | * | * |

| Author | Results | | | | | | | | | | Total | Quality |
| --- | --- | --- | --- | --- | --- | --- | --- | --- | --- | --- | --- | --- |
|  | Participant flow (a diagram is strongly recommended) | | Recruitment | | Baseline data | Numbers analyzed | Outcomes and estimation | | Ancillary analyses | Harms |  |  |
|  | a | b | a | b |  |  | a | b |  |  |  |  |
| Arntzen et al. (2020) | * | * | * | * | * | * | * | * | * | - | 24 | High |
| Nasseri et al. (2020) | * | * | * | - | * | * | * | * | * | - | 19 | Moderate |
| Plow et al. (2019) | * | * | - | * | * | * | * | * | * | - | 20 | Moderate |
| Motl et al. (2017) | * | - | - | - | * | * | * | * | * | - | 18 | Moderate |
| Paul et al. (2019) | * | * | * | * | * | * | * | * | * | * | 21 | High |
| Motl et al. (2023) | * | * | * | * | * | * | * | * | * | - | 21 | High |
| Learmonth et al. (2017) | * | * | * | * | * | * | * | * | * | - | 20 | Moderate |
| Pilutti et al. (2014) | * | * | * | * | * | * | * | * | * | - | 20 | Moderate |

Supplementary Figure 1. Bubble plots of meta-regression results

|  |  |
| --- | --- |
|  | Insufficient data for Moderate to vigorous for BMI |
|  |  |
|  |  |

Supplementary figure 2. Summary of risk of bias results evaluated:

(A) The risk of bias levels of key RCT components rated in every included study

| Author, date | Bias in Random sequence generation | Bias in Allocation concealment | Bias in Blinding of participants and personnel | Bias in Blinding of outcome assessment | Bias in Incomplete outcome data | Bias in Selective outcome  reporting |
| --- | --- | --- | --- | --- | --- | --- |
| Arntzen et al. (2020) | 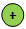 | 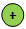 | 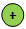 | 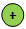 | 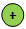 | 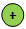 |
| Nasseri et al. (2020) | 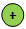 | 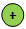 | 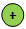 | 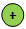 | 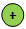 | 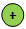 |
| Plow et al. (2019) | 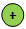 | 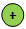 | 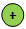 | 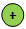 | 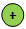 | 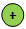 |
| Motl et al. (2017) | 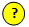 | 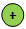 | 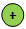 | 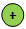 | 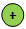 | 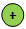 |
| Paul et al. (2019) | 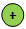 | 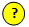 | 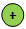 | 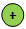 | 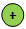 | 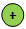 |
| Motl et al. (2023) | 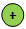 | 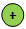 | 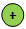 | 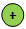 | 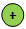 | 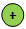 |
| Learmonth et al. (2017) | 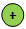 | 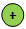 | 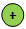 | 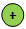 | 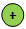 | 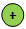 |
| Pilutti et al. (2014) | 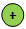 | 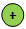 | 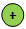 | 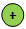 | 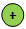 | 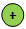 |
| 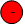 Yes (high risk of bias) 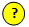 Unclear 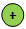 No (low risk of bias)  **RCT** | | | | | | |

(B) the percentage of each risk of bias level of the key components.
